# Identification of Potential Bioactive Ingredients and Mechanisms of the Guanxin Suhe Pill on Angina Pectoris by Integrating Network Pharmacology and Molecular Docking

**DOI:** 10.1155/2021/4280482

**Published:** 2021-08-11

**Authors:** Mingmin Wang, Shuangjie Yang, Mingyan Shao, Qian Zhang, Xiaoping Wang, Linghui Lu, Sheng Gao, Yong Wang, Wei Wang

**Affiliations:** ^1^Dongzhimen Hospital, Beijing University of Chinese Medicine, Beijing 100700, China; ^2^School of Chinese Medicine, Beijing University of Chinese Medicine, Beijing 100029, China; ^3^School of Life Sciences, Beijing University of Chinese Medicine, Beijing 100029, China; ^4^Department of Gastroenterology, The Shanghai Tenth People's Hospital, Tongji University, Shanghai 200092, China

## Abstract

The Guanxin Suhe pill (GSP), a traditional Chinese medicine, has been widely used to treat angina pectoris (AP) in Chinese clinical practice. However, research on the bioactive ingredients and underlying mechanisms of GSP in AP remains scarce. In this study, a system pharmacology approach integrating gastrointestinal absorption (GA) evaluation, drug-likeness (DL) evaluation, target exploration, protein-protein-interaction analysis, Gene Ontology (GO) enrichment analysis, network construction, and molecular docking was adopted to explore its potential mechanisms. A total of 481 ingredients from five herbs were collected, and 242 were qualified based on GA and DL evaluation. Target exploration identified 107 shared targets between GSP and AP. Protein-protein interaction identified VEGFA (vascular endothelial growth factor A), TNF (tumor necrosis factor), CCL2 (C-C motif chemokine ligand 2), FN1 (fibronectin 1), MMP9 (matrix metallopeptidase 9), PTGS2 (prostaglandin-endoperoxide synthase 2), IL10 (interleukin 10), CXCL8 (C-X-C motif chemokine ligand 8), IL6 (interleukin 6), and INS (insulin) as hub targets for GSP, which were involved in the inflammatory process, ECM proteolysis, glucose metabolism, and lipid metabolism. GO enrichment identified top pathways in the biological processes, molecular functions, and cell components, explaining GSP's potential AP treatment mechanism. Positive regulation of the nitric oxide biosynthetic process and the response to hypoxia ranked highest of the biological processes; core targets that GSP can regulate in these two pathways were PTGS2 and NOS2, respectively. Molecular docking verified the interactions between the core genes in the pathway and the active ingredients. The study lays a foundation for further experimental research and clinical application.

## 1. Introduction

Angina pectoris (AP) is chest pain or discomfort which often occurs with stimulating factors, like physical activity or emotional stress. There is accumulating evidence to suggest that AP is associated with myocardial ischemia and coronary atherosclerosis [1]. Frequently used antianginal drug therapy includes organic nitrates, *β*-blockers, calcium channel blockers, and nicorandil [2]. These medications mainly control symptoms of myocardial ischemia by reducing myocardial oxygen demand and increasing coronary blood flow. However, despite these antianginal drug therapies being able to effectively control chest pain symptoms and improve physical exercise tolerance, these drugs have also been reported to induce receptor tolerance because of their regular prescription [3].

Traditional Chinese medicine has been reported to effectively control AP. For instance, *Panax notoginseng* can reduce cardiovascular events, alleviate AP symptoms, and reduce the attack frequency of AP [4]. Acupuncture can also safely and effectively improve physical restrictions, emotional distress, and attack frequency in patients with stable AP [5]. In addition, Suxiao Jiuxin Wan is effective in treating AP with no severe side effects identified to date [6].

The Guanxin Suhe pill (GSP) is a traditional Chinese medicine formula for AP in Chinese Pharmacopoeia. It includes five herb components: *Styrax* (Storax, Suhexiang), *Borneolum Syntheticum* (Borneol, Bingpian), *Resi oliani* (Frankincense, Ruxiang), *Lignum Santali Albi* (Sandalwood, Tanxiang), and *Inula helenium* (Elecampane Inula, Tumuxiang). The GSP and its components have been proved to have an antianginal effect [7]. A clinical trial on 120 patients with cold obstruction causing “qi stagnation” syndrome was conducted to determine whether GSP increases the effect of isosorbide mononitrate. The result found that GSP can significantly relieve the symptoms of AP, including the frequency and duration of angina attacks [8]. However, the mechanism of GSP on AP has not been thoroughly investigated.

Network pharmacology is becoming a cutting-edge research field in drug discovery and development [9]. By integrating reductionist and systems approaches and computational and experimental methods, network pharmacology studies emphasize the paradigm shift from “one target, one drug” to “network target, multicomponent therapeutics” [10]. The multi-ingredient and multitarget nature of Chinese medicine makes it an ideal field for network pharmacology [11].

Molecular docking can predict ligand-target interaction at a molecular level [12]. As an established structure-based *in silico* simulation assay, molecular docking has been widely used in the drug discovery field [13]. The experimental screening of large libraries of compounds against molecular target panels, that is, high-throughput screening (HTS), has been recognized as the gold standard in biology discovery. However, the high cost of the experimental screening remains a drawback. Docking enables researchers to virtually screen databases of approved drugs, natural products, or synthesized compounds into a group of biological targets of interest within a reasonable time [14]. Virtual screening and target profiling have made molecular docking a novel approach for active ingredient screening and mechanism deciphering in Chinese medicine research.

Here, we took advantage of the most comprehensive traditional Chinese medicine (TCM) database to date, the HERB database (a high-throughput experiment- and reference-guided database of TCM), to explore the core ingredients and targets of GSP in treating AP. Next, a systematic pharmacological method integrating ADME (absorption, distribution, metabolism, and excretion) screening, network pharmacology, and molecular docking was used to elucidate the underlying mechanism of the active ingredients in GSP for AP treatment. It is anticipated that the study will promote future studies in TCM, with the concomitant development of more effective therapeutic remedies for AP.

## 2. Materials and Methods

### 2.1. Identification of Chemical Ingredients in GSP

According to the 2015 edition of Chinese Pharmacopoeia, GSP included five herbs: *Styrax* (Storax, Suhexiang), *Borneolum Syntheticum* (Borneol, Bingpian), *Resi oliani* (Frankincense, Ruxiang), *Lignum Santali Albi* (Sandalwood, Tanxiang), and *Inula helenium* (Elecampane Inula, Tumuxiang) (Table 1). We searched the HERB database (a high-throughput experiment- and reference-guided database of TCM) (http://herb.ac.cn.) for the ingredients of the five herbs in GSP. HERB integrates multiple TCM databases to construct a list of TCM herbs and ingredients [15]. Several widely used TCM databases such as SymMap, TCMID 2.0, TCMSP 2.3, and HIT were included [16–19]. To date, the HERB database is the most comprehensive database for Chinese medicine ingredients. This database was interrogated to get a complete view of the known chemical ingredients of GSP. Chemical features of the ingredients were downloaded for the next step in the analysis.

### 2.2. ADME Screened Ingredients with Gastrointestinal Absorption (GA) and Drug-Likeness (DL) Prediction

Pharmacokinetic parameters like bioavailability and DL are as crucial in a small molecule as an effective ingredient. Since GSP is orally administered, we considered GA a necessary pharmacokinetic behavior for active ingredient evaluation. The BOILED-Egg model (the Brain or Intestinal Estimate D permeation method) was applied to determine GA [20].

DL, which is established from structural or physicochemical inspections of oral drug-candidate compounds, assesses qualitatively the chance for an ingredient to become an oral drug according to bioavailability [21]. Five methods were applied as filters for DL evaluation, that is, the Lipinski (Pfizer), Ghose (Amgen), Veber (GSK), Egan (Pharmacia), and Muegge (Bayer) methods [22–26]. All the above methods are integrated into an online tool named SwissADME (http://www.swissadme.ch/) [27]. According to the PubChem identification of ingredients provided by HERB, we searched the simplified molecular-input line-entry system (SMILES) in PubChem (https://pubchem.ncbi.nlm.nih.gov/) and uploaded the SMILES onto the SwissADME website. Chemicals were screened by the following criteria: if the prediction results of the component were both “high” GA and “yes” in more than two of the five filters in the DL prediction, it met our inclusion criteria and progressed to the next screening step.

### 2.3. Common Targets of GSP Ingredients in AP

#### 2.3.1. Potential Therapeutic Targets of GSP Active Ingredients

The HERB database was used to acquire potential therapeutic targets of GSP active ingredients. Apart from database mining targets in the TCM databases such as TCMSP, SymMap, HIT, and TCMID, as mentioned above, HERB also integrates the functional module named “reference mining,” which contains target information from manually curated reference data from 17886 references. HERB identities of active ingredients were uploaded to the database to retrieve them according to potential therapeutic targets.

#### 2.3.2. Prediction of Known Therapeutic Targets for AP

To establish a known therapeutic target dataset of AP, several disease target databases were utilized, including DisGeNET (https://www.disgenet.org/), GeneCards (https://www.genecards.org/), OMIM (https://www.omim.org/), PharmGKB (https://www.pharmgkb.org/), and Therapeutic Target Database (http://db.idrblab.net/ttd/) [28–32]. The keyword “angina pectoris” was used to search the candidate targets. Since GeneCards is a database with enormous web-based, deep-linked cards for each of the >73000 human gene entries, its disease target prediction result contains thousands of low-relevance targets. To eliminate the native effect of low-relevance targets, targets from GeneCards with relevance scores <10 were excluded. Disease targets from all other databases were applied with no exclusion. Targets recruited from GeneCards, along with all the targets from the other four databases, were pooled and deduplicated to establish a disease target list.

#### 2.3.3. Identification of Intersection Target of Ingredients and AP

Potential therapeutic targets of GSP ingredients and recruited known therapeutic targets for AP were uploaded to Gene Venn (http://genevenn.sourceforge.net/) [33]. This online tool was used to find out the overlap targets of the HERB and disease-related targets. The protein names of ingredient and disease targets were entered in the text areas on the initial welcome page and processed. The server processed the target lists and created a Venn diagram, which showed the intersection target list of two groups and the unique targets of each group.

### 2.4. Gene Ontology (GO) Pathway Enrichment

The GO project provides an ontology of defined terms representing gene product properties. GO covers three domains: cellular components, molecular function, and biological processes. Intersection targets of ingredients and disease were uploaded to DAVID (https://david.ncifcrf.gov/) for functional annotation of the herbs' targets [34]. In addition, GO pathway enrichment was performed for the biological processes, cell components, and molecular functions.

### 2.5. Network Construction

The possible protein-protein interactions (PPIs) were acquired from the STRING database (https://string-db.org/), which covered almost all the known functional interactions between the expressed proteins [35]. Moreover, PPI network pairs with overall combined scores above 0.4 were included. The combined results of PPIs were imported to CytoSpace software (version 3.7.1, Boston, MA, USA), and the PPI network was reconstructed with the “CytoHubba” plugin. The degrees of freedom in a topology network reflect the strength of a node's connection with other nodes in the network. A high degree value in the PPI network indicates a high number of node edges. Thus, high targets with high degree values are more likely to play an essential role in regulation. Ten targets with the highest degree value were identified as hub targets and used to construct a hub-target network. The interactions of herb-ingredient-target networks for top-10 hub targets and two top-ranked biological process pathways were visualized using CytoSpace software.

### 2.6. Molecular Docking of Most Targeted Proteins in the Top Pathways

In the herb-ingredient-target network of the specific, enriched pathway, the target with the most connected ingredients was identified as the core target in the pathway. Molecular docking was used to assess interactions between ingredients and core targets in the top-ranked biological process pathways. The three-dimensional (3D) structures of chemicals were downloaded from TCMSP (https://tcmspw.com/tcmsp.php) [18]. Protein 3D crystal structures were downloaded from the RCSB Protein Data Bank (http://www.rcsb.org) [36].

AutoDock Tools v1.5.6 was used to open the structure, add the nonpolar hydrogen, calculate Gasteiger charges of the molecule, and add the nonpolar hydrogen for each ligand [37]. The exhaustiveness was set as 20. All the other parameters were set as default. AutoDock Vina 1.1.2 was used to conduct semiflexible docking [38]. The docking conformation with the strongest affinity was adopted as the final docking conformation. Docking conformation with the lowest binding energy was used for analysis. The top-five ingredients with strong binding to each target were selected. PyMol was used to draw the binding graphs [37].

## 3. Results

### 3.1. Workflow

**Figure 1:**
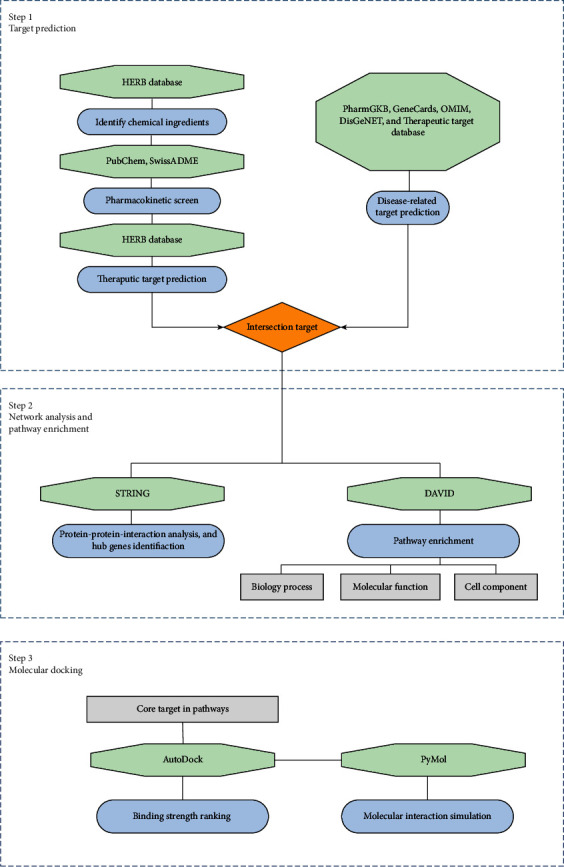
Workflow diagram of the present study.

The specific workflow is depicted in Figure 1. Firstly, the chemical ingredients of the five components in GSP were searched for in the database, and the active ingredients were screened out by pharmacokinetics, including GA and DL. Next, the targets of active ingredients from GSP and the targets of AP were retrieved from multiple databases. Overlap targets were acquired from Gene Venn diagrams. Subsequently, common targets of ingredient and disease targets were uploaded for possible PPIs and GO analyses. PPI network and herb-ingredient-target networks were constructed. Core targets of top pathways were applied for molecular docking screens with interacted ingredients. PyMol software was used to conduct interaction simulations of core targets and high-ranked ingredients.

### 3.2. Candidate Active Ingredients in GSP

After searching HERB, a total of 481 compounds were collected, including 123, 75, 170, 134, and 14 compounds in *Styrax* (Storax, Suhexiang), *Borneolum Syntheticum* (Borneol, Bingpian), *Resi oliani* (Frankincense, Ruxiang), *Lignum Santali Albi* (Sandalwood, Tanxiang), and *Inula helenium* (Elecampane Inula, Tumuxiang), respectively (Supplementary Table 1). According to the PubChem identification provided by the HERB database, the SMILE structure of every component was collected from PubChem.

### 3.3. Ingredient Screening for High GA and Good DL

The SMILE structure of ingredients was then uploaded to SwissADME, and the ingredients were screened according to the GA and DL by the criteria mentioned in the methods. After deduplication, 242 components were qualified, including 52, 30, 85, 80, and 7 in *Styrax* (Storax, Suhexiang), *Borneolum Syntheticum* (Borneol, Bingpian), *Resi oliani* (Frankincense, Ruxiang), *Lignum Santali Albi* (Sandalwood, Tanxiang), and *Inula helenium* (Elecampane Inula, Tumuxiang), respectively (Supplementary Table 1). Several qualified components were presented in more than one herb. All ingredients qualified by GA and DL were adopted to screen intersection targets with AP.

### 3.4. Intersection Targets of GSP Ingredients and AP

**Figure 2:**
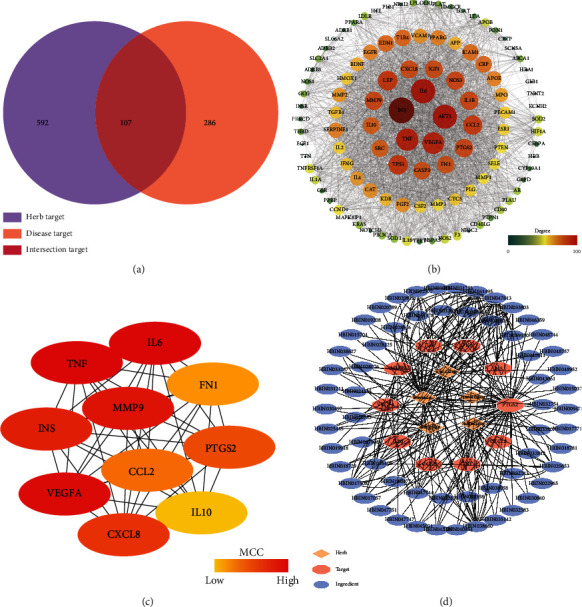
Intersection target genes and protein-protein interactions (PPIs) network of top-10 hub targets. (a) Intersecting target genes between targets of GSP and AP; (b) PPIs network of intersecting targets; (c) PPI network of top-10 hub targets; (d) herb-ingredient-target network of top-10 targets.

**Figure 3:**
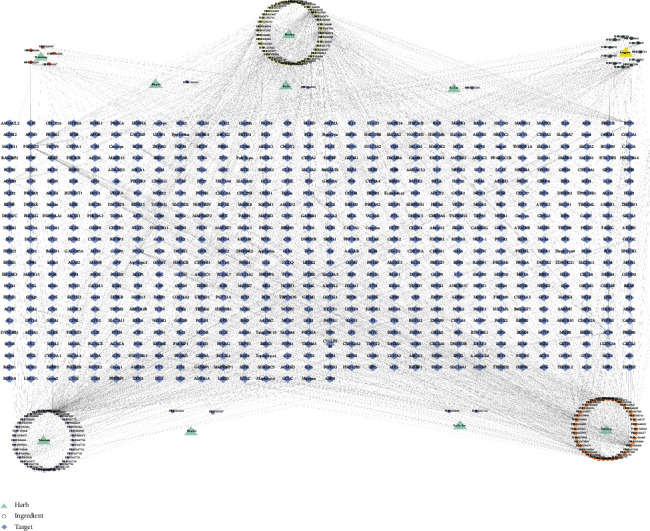
Herb-ingredient-target network of GSP for angina pectoris.

After deduplication, 699 related targets of GSP were collected from HERB. Meanwhile, 393 AP-related targets were collected from OMIM, DisGeNET, GeneCards, OMIM, PharmGKB, and TTD. Among the intersection of GSP and AP targets, there were 107 shared targets adopted for PPI analysis in STRING (Figure 2(a)). To provide a general view of interactions between herbs, ingredients, and AP-related targets, an herb-ingredient-target network was constructed for these 107 intersection targets (Figure 3).

### 3.5. PPIs and Pathway Functional Enrichment

#### 3.5.1. Possible PPIs in Intersection Targets

The PPI results of 107 intersection targets derived from STRING were imported to CytoSpace to construct a topology network with the “CytoHubba” plugin (Figure 2(b)). The intersection targets were ranked according to the degree value of the topology network. Among them, ten targets including VEGFA (vascular endothelial growth factor A), TNF (tumor necrosis factor), CCL2 (C-C motif chemokine ligand 2), FN1 (fibronectin 1), MMP9 (matrix metallopeptidase 9), PTGS2 (prostaglandin-endoperoxide synthase 2), IL10 (interleukin 10), CXCL8 (C-X-C motif chemokine ligand 8), IL6 (interleukin 6), and INS (insulin) were ranked highest using Maximal Clique Centrality. These ten targets were recognized as hub targets. CytoSpace software was used to construct the PPI networks and herb-ingredient-target networks (Figures 2(c) and 2(d)).

#### 3.5.2. GO Pathway Enrichment

**Figure 4:**
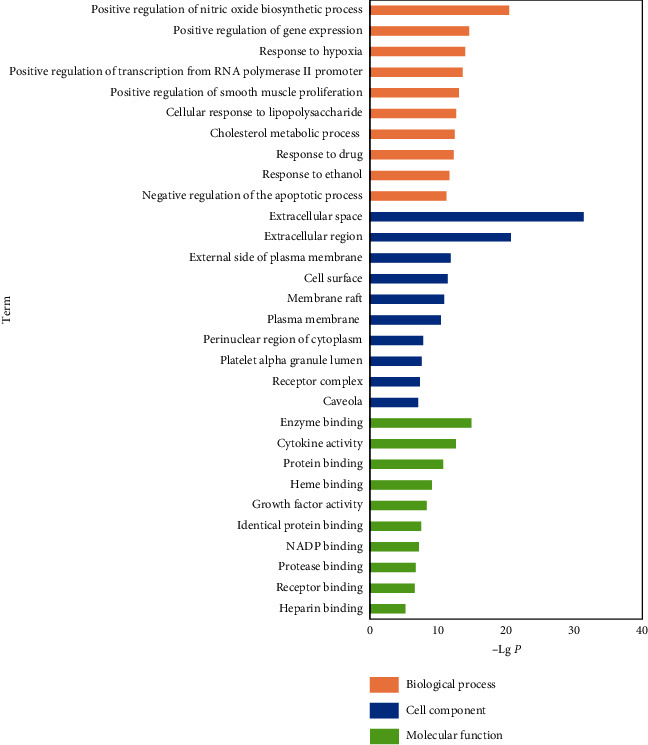
Gene ontology pathway enrichment. Top-10 pathways in BP, MF, and CC according to −Log *P*.

**Figure 5:**
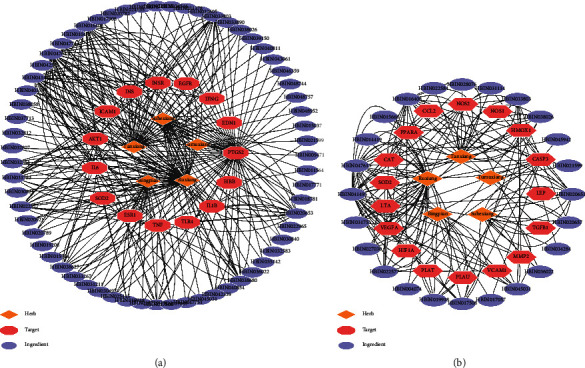
Herb-ingredient-target network of top pathways. (a) Herb-ingredient-target network of GO:0045429 (positive regulation of nitric oxide biosynthetic process) related target; (b) herb-ingredient-target network of GO:0001666 (response to hypoxia) related target.

The GO enrichment analysis of the 107 common targets was analyzed with DAVID. All the biological processes (BP), molecular functions (MF), and cell component (CC) pathways obtained by GO enrichment were ranked using −Log*P* (Figure 4). In the biological process GO enrichment, several AP and coronary atherosclerosis-related pathways, such as the positive regulation of nitric oxide biosynthetic process, ranked top according to the −Log*P* value. The herb-ingredient-target network of the two pathways ranked highest in the biological process as shown in Figure 5. The positive regulation of gene expression ranked second in the biological processes. Since this process is very general and does not clearly provide an insight into the mechanism of the disease, this pathway was omitted in further analyses.

### 3.6. Molecular Docking of the Most Targeted Proteins in the Top Pathways

**Figure 6:**
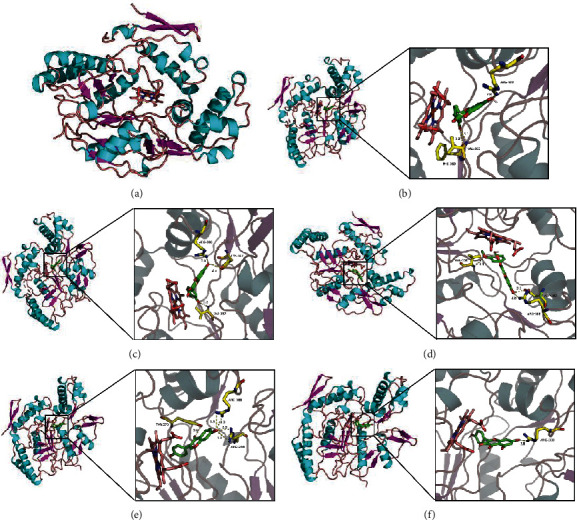
Molecular docking between NOS2 and top-five ingredients. (a) Protein structure of NOS2 (PDB ID:4cx7). (b) Molecular interaction between MOL000006 luteolin and NOS2. (c) Molecular interaction between MOL000098 quercetin and NOS2. (d) Molecular interaction between MOL000354 isorhamnetin and NOS2. (e) Molecular interaction between MOL000390 daidzein and NOS2. (f) Molecular interaction between MOL000422 kaempferol and NOS2.

**Figure 7:**
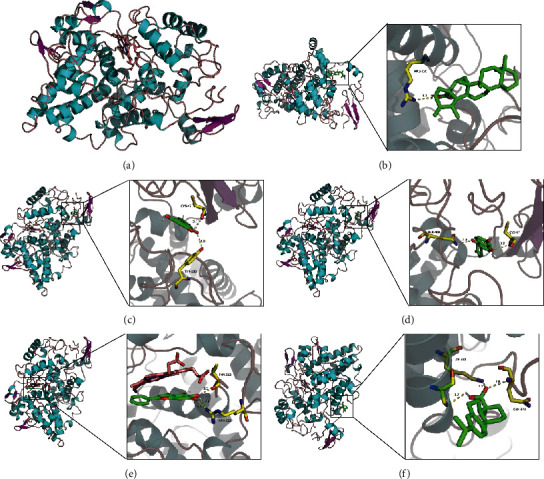
Molecular docking between PTGS2 and top-five ingredients. (a) Protein structure of PTGS2 (PDB ID: 5fdq). (b) Molecular interaction between MOL008498 ursolic acid and PTGS2. (c) Molecular interaction between MOL000008 apigenin and PTGS2. (d) Molecular interaction between MOL000390 daidzein and PTGS2. (e) Molecular interaction between MOL002714 baicalein and PTGS2. (f) Molecular interaction between MOL003782 dehydroabietic acid and PTGS2.

According to the GO pathway enrichment, the positive regulation of nitric oxide biosynthetic process and the response to hypoxia ranked highest in the biological process pathways. PTGS2 in NO and NOS2 in hypoxia demonstrated 54 and 11 compound interactions, respectively, ranking the highest in these two pathways (Supplementary Table 2). Thus, PTGS2 and NOS2 were identified as core targets in these two pathways. We then conducted molecular docking between PTGS2, NOS2, and the corresponding compounds. Interaction scores are shown in Supplementary Tables 3 and 4. The molecular interaction graphs for the top-five ingredients of each component were then constructed using PyMol (Figures 6 and 7). All ten ingredients interacted with corresponding targets mainly through a hydrogen bond. Residual interaction information is shown in Tables 2 and 3.

## 4. Discussion

According to the topology network's degree value, the top-10 hub genes in the PPI network were identified. They were VEGFA, TNF, CCL2, FN1, MMP9, PTGS2, IL10, CXCL8, IL6, and INS. VEGFA is a member of the PDGF/VEGF growth factor family. It induces proliferation and migration of vascular endothelial cells [39]. VEGFA has also been proved to be essential for both physiological and pathological angiogenesis [40]. TNF, CCL2, IL10, and IL6 belong to the cytokine family and are involved in inflammatory processes [41]. FN1 and MMP9 are proteins in the extracellular matrix and are involved in ECM proteolysis [42]. Research has proved that AP shows an imbalanced collagen turnover even without significant obstructive coronary artery disease [43]. PTGS2 is an inducible isozyme responsible for prostanoid biosynthesis and involved in inflammation and mitogenesis [44]. CXCL8, also known as IL-8, is a member of the CXC chemokine family and is a significant mediator of the inflammatory response [45]. INS is a peptide hormone that plays a vital role in the regulation of carbohydrate and lipid metabolism [46].

The function of hub targets mainly focuses on the regulation of inflammation, ECM regulation, and regulation of metabolism. These are vital processes in the pathology of coronary atherosclerosis. Inflammation is an essential pathological process of coronary atherosclerosis, which is the major underlying cause of AP. A critical aspect in managing patients with stable angina is treating underlying coronary atherosclerotic disease and reducing the overall risk burden to prevent future cardiac events and progression of coronary disease. GSP might exert a protective effect on AP through these processes.

In the biological process GO enrichment, several AP and coronary atherosclerotic related pathways ranked top according to the *P* value and gene counts. Positive regulation of the nitric oxide biosynthetic process, response to hypoxia, positive regulation of smooth muscle proliferation, cholesterol metabolic process, negative regulation of the apoptotic process, inflammatory response, and angiogenesis were marked as essential pathways involved in the therapeutic mechanisms of GSP.

Positive regulation of the nitric oxide biosynthetic process ranked highest. Nitric oxide is a vasodilator produced by several cell types [47]. The function of nitric oxide in vessel health is double-edged. On the one hand, endothelial nitric oxide synthase (NOS) produces nitric oxide to maintain the physiological level of NO, which is crucial for vascular endothelial homeostasis, while on the other, different stress-stimulating factors can induce the activation of inducible nitric oxide synthase (iNOS) [48]. NO overproduction induced by the activation of iNOS can lead to endothelial dysfunction and the development of atherosclerosis in the late stages [49]. According to the target prediction of GSP, ingredients in GSP can bind to iNOS and COX2 (PTGS2) to inhibit the overproduction of NO. The inhibition of overproduced NO also can alleviate the inflammation induced by NO.

Nitric oxide can cause vasodilation via its effect on vascular smooth muscle cells. However, the overproduction of NO, which is proinflammatory, is detrimental to cardiovascular disease [50]. PTGS2 has been identified as the core target gene involved in this pathway. PTGS2, also known as COX2, is an important factor in the inflammatory pathway. Research has proved that COX2 also influences the positive regulation of the nitric oxide biosynthetic process [51]. A low dose of COX2 inhibitor has been proved to have a protective effect; the mechanism is based on what is known about the complex biology of cyclooxygenase in different tissue compartments, including the vascular endothelium, myocardium, and atherosclerotic plaques. Ursolic acid, apigenin, daidzein, baicalein, and dehydroabietic acid are the top-five ingredients according to the binding score. Ursolic acid contacts with ARG120, which is a major contributor to both the inhibition and catalysis of PTGS2. The PTGS2 inhibitory properties of other contact residues have also been reported [52].

The response to hypoxia ranked third. AP often involves coronary artery spasm or stenosis, which leads to the disruption of oxygen and nutrient transportation via blood flow [53]. This hemodynamic disorder can lead to heart tissue hypoxia. Hypoxia signaling plays a vital role in cardiac and vascular remodeling in the pathogenesis of cardiovascular diseases [54]. In a hypoxic environment, HIF-*α* protein is stabilized. It binds to the hypoxia-responsive element (HREs) of each target gene, including glucose metabolism (pyruvate dehydrogenase kinase (PDK)), angiogenesis (vascular endothelial growth factor A (VEGFA)), and erythropoiesis (erythropoietin (EPO)) [55, 56]. Thus, the ingredients of GSP may alleviate the cardiac remodeling process through the tissue response to hypoxia.

Response to the hypoxia also ranked top in the biological process analysis. NOS2 is the core target in this pathway. The exact mechanism of angina pain remains unclear, but it is related to a mismatch between myocardial oxygen demand and supply [57]. It has been shown that, after tissue hypoxia, iNOS overexpression plays a vital role in tissue injury [58]. Moreover, hypoxia alters the expression of several transcription factors responsible for iNOS expression, and downregulation of iNOS can limit cell injury caused by hypoxia [59]. Therefore, iNOS inhibition can be a novel therapeutic mechanism for protection from hypoxia-induced injury and cell death. Luteolin, quercetin, isorhamnetin, daidzein, and kaempferol rank highest according to the molecular docking study. Their contacting residue, mainly on the H4B binding pocket in the oxygenase domain, is essential for H4B cofactor binding [60]. These ingredients might contact this domain and thus influence the subsequent dimerization and function of the enzyme.

Our results provide a potential binding model for the top-ranked ingredients of NOS2 and PTGS2. As mentioned above, NOS2 inhibitors and PTGS2 inhibitors have been identified for the treatment of cardiovascular disease. However, the selective inhibitors have vast side effects. The multitarget and multi-ingredient nature of Chinese medicine provides another avenue for drug development from Chinese medicine.

## 5. Conclusions

This study identifies the potential bioactive ingredients, biological targets, and functional pathways of GSP for AP, which reveals the characteristics of multiple ingredients, targets, pathways, and mechanisms of GSP. To the best of our knowledge, it is the first study that integrates network pharmacology and molecular docking to predict the bioactive ingredients and mechanisms of GSP in the treatment of AP. The study lays a foundation for further experimental research and clinical application.

However, the current study also possesses limitations. Although the potential bioactive ingredients and mechanisms of GSP against AP have been expounded by integrating system pharmacology and molecular docking, further experimental verification for compounds and mechanisms predicted by the network analysis is lacking and is a drawback of the present study. To confirm the functional and pharmacological mechanisms of the major bioactive ingredients identified, more pharmacological experiments and *in vitro* molecular binding assays will be conducted in the near future.

## Figures and Tables

**Table 1:** Information of herbs in GSP.

No.	Name			Use part	Properties	Number		Abbreviation
	Latin	English	Chinese pinyin			Components	Candidate compounds	
1	*Styrax*	Storax	Suhexiang	Balsam from trunk	Warm; pungent	123	52	SHX
2	*Borneolum Syntheticum*	Borneol	Bingpian	Resin	Minor cold; pungent; bitter	75	30	BP
3	*Resi oliani*	Frankincense	Ruxiang	Balsam	Warm; pungent; bitter	170	85	RX
4	*Lignum Santali Albi*	Sandalwood	Tanxiang	Heartwood	Warm; pungent	134	80	TX
5	*Inula helenium*	Elecampane Inula	Tumuxiang	Root	Warm; pungent; bitter	14	7	TMX

**Table 2:** Molecular binding information of PTGS2 and top-5 ingredients.

Target	Compound	TCMSP ID	Contacting residue	Binding distance	Score
PTGS2	Ursolic acid	MOL008498	ARG120	3.1	−9.6

PTGS2	Apigenin	MOL000008	TYR130	3	−9
			CYS47	2.1	

PTGS2	Daidzein	MOL000390	GLN461	2.9	−8.4
			CYS47	3.2	

PTGS2	Baicalein	MOL002714	THR212	3.2	−7.5
			ARG222	2.8, 3.1	

PTGS2	Dehydroabietic acid	MOL003782	SER121	2.7	−7.5
			LYS532	3.1	
			GLN372	2.8	

**Table 3:** Molecular binding information of NOS2 and top-5 ingredients.

Target	Compound	TCMSP ID	Contacting residue	Binding distance	Score
NOS2	Luteolin	MOL000006	ARG388	2.8	−9.5
			VAL352	3.1	
			PHE369	2.8	

NOS2	Quercetin	MOL000098	ARG388	3	−9.3
			TYR347	2.3	
			VAL352	3.2	

NOS2	Isorhamnetin	MOL000354	VAL352	3.4	−8.9
			ASP382	2.3	
			ARG388	2.8	

NOS2	Daidzein	MOL000390	TYR373	3	−8.9
			ARG388	3.3, 3.3	
			ARG266	2.9, 3.4	

NOS2	Kaempferol	MOL000422	ARG388	2.8	−8.9

## Data Availability

All the relevant data of this article are available at https://github.com/MingminW/Guanxinsuhe-Pill.git.

## Supplementary Materials

Supplementary Materials Supplementary Table 1: herb ingredient ID lists and qualified ingredient ID list. Supplementary Table 2: ingredient-target interaction lists of top targets. Supplementary Table 3: interaction scores of PTGS2 and ingredients. Supplementary Table 4: interaction scores of NOS2 and ingredients.
